# Arrhythmia Induced by Positional Change Under General Anesthesia Related to Caudal Movement of the Peripherally Inserted Central Venous Catheter: A Case Report

**DOI:** 10.1155/cria/9185758

**Published:** 2024-12-27

**Authors:** Isao Utsumi, Tomasz Hascilowicz, Yasushi Mio, Sachiko Omi

**Affiliations:** Department of Anesthesiology, The Jikei University School of Medicine, Tokyo, Japan

## Abstract

**Background:** The novel case report of a temporary arrhythmia that developed after a positional change in a patient under general anesthesia emphasizes the possibility of potentially lethal cardiac arrhythmias that may develop owing to caudal movement of the peripherally inserted central venous catheter ([CVC] PICC) tip when changing patient positions.

**Case Presentation:** We present a case of temporary arrhythmia that developed after a positional change in a 44-year-old female patient scheduled for laparoscopic adrenalectomy under general anesthesia. She had undergone preoperative insertion of a PICC using an electrocardiogram (ECG)-guided tip confirmation system (TCS).

**Conclusion:** The case report discusses the optimal TCS and emphasizes vigilant ECG monitoring, suggesting measures to prevent these arrhythmias under general anesthesia.

## 1. Background

Complications, such as arterial puncture, pneumothorax, hemothorax, and infections, are reported to occur less frequently with the placement of peripherally inserted central venous catheters ([CVCs] PICCs) than with the placement of other CVCs [1, 2]. The use of PICC has increased in clinical settings, including during the perioperative period. Consequently, more patients undergo surgery under general anesthesia with PICCs inserted presurgically. PICC use has become more widespread due to newly introduced and more convenient insertion techniques, such as those adopting passive magnet tracking and the electrocardiogram (ECG)-guided tip confirmation system (TCS) [3], which facilitates catheter placement at the bedside without confirmatory imaging, and insertions performed by trained nurse practitioners. It is well known that guidewires used during PICC placement may cause cardiac arrhythmias if they progress too far into the right atrium (RA) [4]. There are limited case reports on positional change–related arrhythmias as the insertion of PICCs is usually performed in awoken patients [5, 6]. Moreover, arrhythmias that occur during general anesthesia and are induced by a catheter inserted with the TCS-guided technique have not been reported.

Arrhythmias related to PICCs are rare. In three cases [5, 6], nonsustained ventricular tachycardia (VT) occurred in awoken patients when their body positions were changed following PICC placement and confirmation of the tip position in the lower superior vena cava (SVC) by fluoroscopy or chest radiography. We presented a case of nonsustained VT that developed after a change in the patient's position under general anesthesia. We also discussed the factors affecting positional change–related arrhythmias in patients with preoperatively inserted PICCs using TCS and suggested possible measures for their prevention and treatment.

## 2. Case Presentation

**Figure 1:**
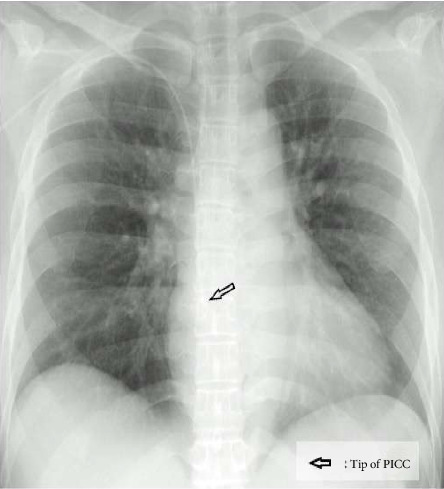
A confirmatory chest radiograph taken after insertion of the peripherally inserted central venous catheter (PICC).

**Figure 2:**
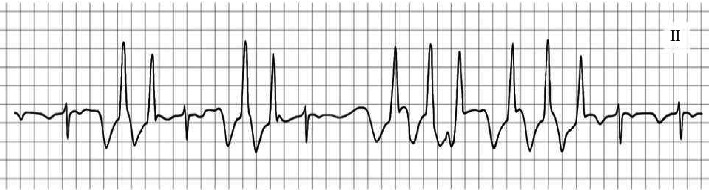
An electrocardiogram (Lead II) showing nonsustained ventricular tachycardia (VT) that occurred during positional change from supine to left lateral decubitus position with adduction and internal rotation of the right arm.

**Figure 3:**
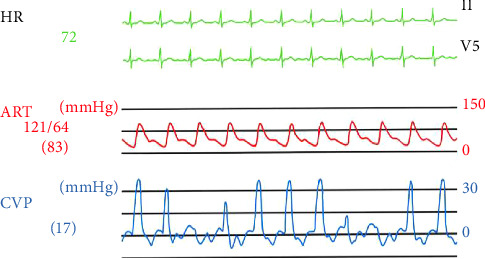
The right atrial and right ventricular pressures measured using PICC lumens during operation. (HR, heart rate; ART, arterial pressure; CVP, central venous pressure).

The patient was a 44-year-old woman (height 155 cm and weight 62 kg) scheduled for laparoscopic right adrenalectomy for primary aldosteronism. She had no previous history of arrhythmia and had not taken any medications that cause arrhythmias. On the day before surgery, a PICC (PowerPICC, Becton, Dickinson and Company, NJ, USA) was inserted via the right brachial–basilic vein and the Sherlock 3CG TCS (Becton, Dickinson and Company, NJ, USA) was used to confirm the position of the catheter tip. It was placed 41 cm from the insertion site based on the maximal *P*-wave on the ECG, observed after confirmation of *P*-wave inversion. The correct position of the tip was then confirmed using chest radiography (Figure 1). After induction of anesthesia, when the patient was placed in the left lateral decubitus position with adduction followed by internal rotation of her right shoulder and flexion of the elbow, monomorphic nonsustained VT occurred (Figure 2). With further, almost maximal, internal rotation of the shoulder, the arrhythmia ceased, and the surgery was commenced as planned. Intraoperatively, the right atrium (RA) and right ventricle (RV) pressures were measured using a PICC lumen (Figure 3). The data indicated respiration-related movements of the catheter in the RV; however, no further arrhythmias were observed. When the patient was returned to the supine position after surgery, arrhythmia did not occur, and the postoperative course was uneventful.

## 3. Discussion

“The Practical Guidelines for Safe CVC Placement and Management” issued by the Japanese Society of Anesthesiologists (2017) recommend Zone *B*, an area around the junction of the left and right innominate veins and the upper SVC, as the optimal position of the catheter tip following insertion [7, 8]; these guidelines pertain to all CVCs and do not particularly focus on PICC tips. The European Society for Clinical Nutrition and Metabolism guidelines (2009) also pertain to all CVCs, presumably including PICCs, and accordingly, the adequate site for catheter placement is when “the tip is in the lower third of the SVC, at the cavoatrial junction (CAJ), or in the upper portion of the RA” [9]. Similar recommendations are presented in “Safe Vascular Access (2016)” (the United Kingdom), where the lower SVC or the upper RA position is considered optimal [10]; however, some American authors exclude the upper RA and limit the optimal position to the lower SVC and in the vicinity of the CAJ [11]. Hence, adequate placement of the catheter, as recommended in the Japanese Society of Anesthesiologists guidelines, is slightly lower [3]; however, these guidelines have been established to reduce the frequency of CVC-related complications (vessel or myocardium perforations, thromboses, occlusions, catheter-related infections, pneumothorax, and arrhythmias) and are based on reported data where CVC tip positions were confirmed either with fluoroscopy or portable chest radiography.

With technological advancements in magnetic tracking and intracavity ECG, PICCs have recently been inserted at the bedside without postinsertion imaging. PICCs placed with the Sherlock 3CG TCS, where the tip position at the CAJ is considered correct according to the observed inversion of the electrocardiographic *P*-wave (NICE Medical Technology Guidance), tend to be inserted too far into the RA. Malpositioning of the catheter tip with the Sherlock 3CG occurs in 56.1% or 20.5% of patients, depending on the definition of the adequate tip position (low SVC/CAJ or mid SVC/low SVC/CAJ/high RA, respectively) [12]. According to Johnston et al., malpositioning might be due to specific characteristics of the 3CG technology (targeting the CAJ, which is sometimes impossible to achieve in clinical settings), difficulty in defining the CAJ position on chest radiographs, and/or inconsistent CVC placement guidelines [12]. Thus, even if the catheter is inserted and fixed in an optimal position, complications may still occur.

In our case, PICC was inserted with the Sherlock 3CG TCS one day before surgery, and arrhythmia did not occur until the patient was positioned for the operation. Movements of the catheter tip (caudally up to 5.3 cm [or 2.2 rib spaces]) [13, 14] due to shoulder adduction have been reported, but only rarely induce VTs. The catheter tips constantly move according to the blood/injection fluid flow changes in the vicinity of the CAJ (lower risk of thrombosis) [12], or the upper RA might occasionally contact the atrioventricular (AV) node/RV wall; however, if this contact is only momentary, arrhythmia will presumably not occur. In contrast, in the lateral decubitus or prone position, contact with the AV node/RV wall [6] may be sufficient to induce VT, which is not observed immediately after PICC insertion (usually in the supine position). A minor change in the body position (in our case, further adduction of the shoulder) might terminate the arrhythmia, but respiration- and blood flow–related movements would not cease (Figure 3). In an awoken patient, arrhythmia may produce symptoms such as shortness of breath, chest pain, or palpitations, and thus provoke a timely response [5, 6]. In contrast, in patients under general anesthesia, suspicion and vigilant observation of the ECG tracing when changing body position remain the only means of early recognition and treatment of PICC-induced arrhythmias. Simulation of the intraoperative position immediately after PICC insertion might allow detection of positional change–induced arrhythmias and prompt early correction of the catheter position (usually pulling back the catheter); however, this is not always feasible in clinical settings.

Notwithstanding inconsistencies in CVC placement guidelines [7, 9, 10], TCS-guided PICC insertions seem to produce fewer instances of tip malpositioning and PICC-related complications. However, PICCs inserted with the Sherlock 3CG TCS tend to be extended further into the RA or RV than in conventionally placed PICCs under fluoroscopic or radiographic guidance and might induce arrhythmias on changing the patient's position. In patients under general anesthesia, such arrhythmias should be suspected and vigilant ECG monitoring should be performed. Placing the patient in the final intraoperative position immediately after PICC insertion may be reasonable, but further studies are required to assess the validity of its routine implementation.

## Data Availability

The data that support the findings of this study are openly available in Authorea at https://doi.org/10.22541/au.161582802.20417989/v1 (DOI: 10.22541/au.161582802.20417989/v1) [15].

## Nomenclature

ART: Artery pressure
AV: *Atrioventricular*
CAJ: Cavoatrial junction
CVC: Central venous catheter
CVP: Central venous pressure
ECG: Electrocardiogram
HR: Heart rate
PICC: Peripherally inserted central venous catheter
RA: Right atrium
RV: Right ventricle
SVC: Superior vena cava
TCS: Tip confirmation system
VT: Ventricular tachycardia
